# (4*S*,5*S*,6*S*)-4-Hy­droxy-3-meth­oxy-5-methyl-5,6-ep­oxy­cyclo­hex-2-en-1-one

**DOI:** 10.1107/S1600536810030850

**Published:** 2010-08-11

**Authors:** Srinuan Tansuwan, Porntana Chanaprat, Thapong Teerawatananond, Nongnuj Muangsin, Surachai Pornpakakul

**Affiliations:** a Research Centre of Bioorganic Chemistry, Department of Chemistry, Faculty of Science, Chulalongkorn University, Bangkok 10330, Thailand

## Abstract

The title compound, C_8_H_10_O_4_, was isolated from culture extracts of the endophytic fungus *Xylaria sp.* (PB-30). The cyclo­hexenone ring exhibits a flattened boat conformation. In the crystal structure, mol­ecules related by translation along the *b* axis are linked into chains through O—H⋯O hydrogen bonds. Weak non-classical C—H⋯O contacts are also observed in the structure.

## Related literature

For background to the structures of bioactive secondary metabolites from endophytic fungus and their activities, see: Tansuwan *et al.* (2007 ▶); Shiono *et al.* (2005 ▶); Mitsui *et al.* (2004 ▶). For related structures and the assignment of the absolute configuration, see: Mitsui *et al.* (2004 ▶); Shiono *et al.* (2005 ▶). For puckering parameters, see: Cremer & Pople (1975 ▶).
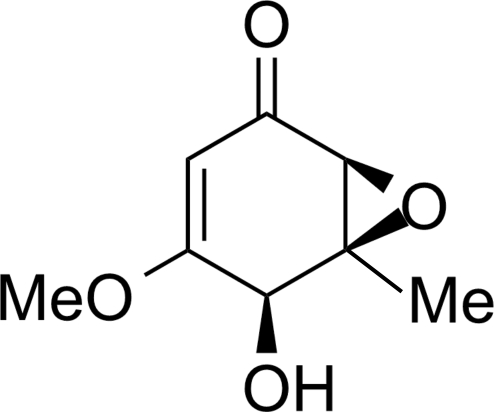


## Experimental

### 

#### Crystal data

C_8_H_10_O_4_
*M* *_r_* = 170.16
Orthorhombic, 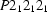

*a* = 4.2208 (1) Å
*b* = 7.5459 (3) Å
*c* = 25.0802 (8) Å
*V* = 798.80 (4) Å^3^
*Z* = 4
Mo *K*α radiation
μ = 0.11 mm^−1^
*T* = 293 K
0.42 × 0.40 × 0.30 mm

#### Data collection

Bruker SMART APEXII CCD area-detector diffractometer
6038 measured reflections
1768 independent reflections
1551 reflections with *I* > 2σ(*I*)
*R*_int_ = 0.021

#### Refinement

*R*[*F* ^2^ > 2σ(*F* ^2^)] = 0.041
*wR*(*F* ^2^) = 0.116
*S* = 1.07
1768 reflections
112 parameters
H-atom parameters constrained
Δρ_max_ = 0.32 e Å^−3^
Δρ_min_ = −0.24 e Å^−3^

### 

Data collection: *APEX2* (Bruker, 2008 ▶); cell refinement: *SAINT* (Bruker, 2008 ▶); data reduction: *SAINT*; program(s) used to solve structure: *SHELXS97* (Sheldrick, 2008 ▶); program(s) used to refine structure: *SHELXL97* (Sheldrick, 2008 ▶); molecular graphics: *ORTEP-3* (Farrugia, 1997 ▶); software used to prepare material for publication: *publCIF* (Westrip, 2010 ▶).

## Supplementary Material

Crystal structure: contains datablocks global, I. DOI: 10.1107/S1600536810030850/cv2746sup1.cif

Structure factors: contains datablocks I. DOI: 10.1107/S1600536810030850/cv2746Isup2.hkl

Additional supplementary materials:  crystallographic information; 3D view; checkCIF report

## Figures and Tables

**Table 1:** Hydrogen-bond geometry (Å, °)

*D*—H⋯*A*	*D*—H	H⋯*A*	*D*⋯*A*	*D*—H⋯*A*
O2—H2*A*⋯O1^i^	0.82	1.99	2.8148 (17)	180
C4—H4⋯O2^ii^	0.98	2.54	3.521 (2)	176
C6—H6⋯O4^iii^	0.98	2.56	3.5208 (17)	167

Symmetry codes: (i) 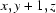; (ii) 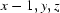; (iii) 
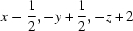
.

**Fig. 1.**
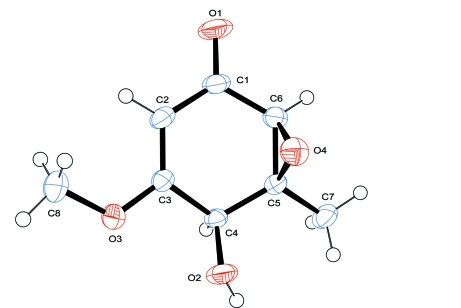
The molecular structure of the title compound, showing 50% probability displacement ellipsoids and the atom numbering scheme. Hydrogen atoms are shown as spheres of arbitrary radius.

## supplementary crystallographic information

### Comment

Endophytic fungi have been proven to be a rich source of novel structural compounds with interesting biological activities and a high level of biodiversity. In the previous investigations of bioactive compounds produced by an endophytic fungus, *Xylaria sp* (strain PB-30), two antimalarial benzoquinones were isolated (Tansuwan *et al.*, 2007). As a part of our continuing search for anticancer metabolites of this fungus, we found that the title compound is one of major metabolite  showing cytotoxicity against various human cell lines, for example breast ductal carcinoma (BT474), human undifferentiated lung carcinoma (CHAGO), human liver hepatoblastoma (HEP-G2), human gastric carcinoma (KATO-3), and human colon adenocarcinoma (SW620) at IC_50_ values of 10.51, 11.11, 6.25, 5.61 and 5.31 µg/ml, respectively.

The title compound was previously isolated from the organic extracts of the fungus xylariaceous endophytic fungus (strain YUA-026), and elucidated on the basis of spectroscopic analysis (Shiono *et al.*, 2005). Herein we present the crystal structure of the title compound, which was isolated from the fermentation culture of the endophytic fungus *Xylaria sp* (strain PB-30) at room temperature under a static condition.

The cyclohexenone ring exhibits a flattened boat conformation with puckering amplitudes *Q* = 0.232 (1), φ = 176.5 (3)° and θ = 81.8°. The 3-methoxy substitutent is in the plane of the cyclohexenone ring to which it is attached [dihedral angle = 7.4 (2)°]. Epoxide ring makes dihedral angles of 87.23 (6)° with the cyclohexenone rings C1—C6. The hydroxyl group locates on the same side of the epoxide ring. In the crystal, the molecules are linked to each other through O—H···O hydrogen bonds, building a polymeric chain parallel to the *b*-axis. Weak non-classical C—H···O contacts are also observed in the structure. The absolute configuration could not be determined therefore it was assigned on the basis of literature data (Shiono *et al.*, 2005).

### Experimental

The endophytic fungus *Xylaria sp.* PB-30 was cultivated in 100 ml of malt extract broth (MEB) in a 250 ml flask (*x* 300) under static condition at room temperature for 35 days. The culture was filtered through filter paper (Whatman No.1). The culture broth (23 *L*) was concentrated by rotary evaporator *in vacuo* to give the concentrated broth (1.5 *L*) and then extracted with EtOAc (x5), CH_2_Cl_2_: MeOH (1:1) (x5) and MeOH (x5), respectively. The solvents were evaporated under reduced pressure at 30°C to give EtOAc crude as yellow viscous liquid (29.61 g), CH_2_Cl_2_: MeOH crude as brown viscous liquid (21.45 g) and MeOH crude as brown viscous liquid (4.74 g).

The EtOAc crude extract (29.61 g) was subjected to a column chromatography [Sephadex^TM^ LH-20 (400 g), column diameter 3.6 cm] using 5% dichloromethane in MeOH as eluent. 10 ml of each fraction was collected. The similar fractions were combined on the basis of TLC profile and monitored by UV, iodine vapor and vanillin/H_2_SO_4_ reagent to give 14 combined fractions.

The fractions EB-5 and EB-6 were combined and purified by crystallization from a 1:1 mixture of CH_2_Cl_2_ and acetone to obtain the title compound as colorless crystals (25.8 mg).

m.p. 153–155¯C; [α]_D_^ 20 ^-100^ o^ (c = 0.1, MeOH); λ_max_ (MeOH) (log ε) 260 nm (3.75); HRESIMS *m/z* 363.0579 [2*M*+Na]^+^*calc.* for (C_8_H_10_O_4_)_2_ Na 363.1055 FT—IR (KBr) *v*_max_ (cm^-1^): 3445, 3070, 3033, 2987, 2940, 2917, 2857, 1642, 1605, 1386, 1300, 1253, 1213, 1070, and 1014; ^1^H-NMR δ (CDCl_3_, 400 MHz) 1.64 (3*H*, s, Me), 2.58 (1*H*, d, *J*=6.0 Hz, 4-OH), 3.32 (1*H*, d, *J*=2.0 Hz, 6-H), 3.76 (3*H*, s, OMe), 4.48 (1*H*, d, *J*=6.0 Hz, 4-H) and 5.25 (1*H*, d, *J*= 2.0 Hz, 2-H) p.p.m.; ^13^C-NMR δ (CDCl_3_, 100 MHz) 18.9 (Me), 56.6 (OMe), 59.4 (C-5), 60.5 (C-6), 69.1 (C-4), 98.2 (C-2), 171.3 (C-3) and 193.4 (C-1) p.p.m.

### Refinement

All H atoms were positioned geometrically and treated as riding, with C—H bonding lengths constrained to 0.93 Å (aromatic CH), 0.96 Å (methyl CH_3_), 0.97 Å (methylene CH_2_), and O—H = 0.84 Å, and with *U*_iso_(H) = 1.2*U*eq(CH) or *U*_iso_(H) = 1.5*U*_eq_(CH_3_, methylene C or OH). 1159 Friedel pair were merged before the final refinement. The absolute configuration was assigned on the basis of the known configuration of the starting material as *R*, *S* and *S* for C4, C5 and C6-positions, respectively.

### Crystal data

**Table:**

C_8_H_10_O_4_	*F*(000) = 360
*M**_r_* = 170.16	*D*_x_ = 1.415 Mg m^−^^3^
Orthorhombic, *P*2_1_2_1_2_1_	Mo *K*α radiation, λ = 0.71073 Å
Hall symbol: P 2ac 2ab	Cell parameters from 2855 reflections
*a* = 4.2208 (1) Å	θ = 2.8–32.8°
*b* = 7.5459 (3) Å	µ = 0.11 mm^−^^1^
*c* = 25.0802 (8) Å	*T* = 293 K
*V* = 798.80 (4) Å^3^	Prism, colourless
*Z* = 4	0.42 × 0.40 × 0.30 mm

### Data collection

**Table:**

Bruker SMART APEXII CCD area-detector diffractometer	1551 reflections with *I* > 2σ(*I*)
Radiation source: Mo	*R*_int_ = 0.021
graphite	θ_max_ = 33.1°, θ_min_ = 2.8°
φ and ω scans	*h* = −6→6
6038 measured reflections	*k* = −11→7
1768 independent reflections	*l* = −37→32

### Refinement

**Table:**

Refinement on *F*^2^	0 restraints
Least-squares matrix: full	H-atom parameters constrained
*R*[*F*^2^ > 2σ(*F*^2^)] = 0.041	*w* = 1/[σ^2^(*F*_o_^2^) + (0.0798*P*)^2^ + 0.0232*P*] where *P* = (*F*_o_^2^ + 2*F*_c_^2^)/3
*wR*(*F*^2^) = 0.116	(Δ/σ)_max_ = 0.001
*S* = 1.07	Δρ_max_ = 0.32 e Å^−^^3^
1768 reflections	Δρ_min_ = −0.24 e Å^−^^3^
112 parameters	

### Special details

**Table:**

Geometry. All e.s.d.'s (except the e.s.d. in the dihedral angle between two l.s. planes) are estimated using the full covariance matrix. The cell e.s.d.'s are taken into account individually in the estimation of e.s.d.'s in distances, angles and torsion angles; correlations between e.s.d.'s in cell parameters are only used when they are defined by crystal symmetry. An approximate (isotropic) treatment of cell e.s.d.'s is used for estimating e.s.d.'s involving l.s. planes.

### Fractional atomic coordinates and isotropic or equivalent isotropic displacement parameters (Å^2^)

**Table:**

	*x*	*y*	*z*	*U*_iso_*/*U*_eq_	
C1	0.6030 (4)	0.12154 (17)	0.88766 (6)	0.0349 (3)	
C2	0.6543 (4)	0.19049 (16)	0.83465 (5)	0.0337 (3)	
H2	0.6778	0.1121	0.8063	0.04*	
C3	0.6687 (3)	0.36664 (16)	0.82548 (5)	0.0268 (2)	
C4	0.6073 (3)	0.50632 (15)	0.86741 (5)	0.0248 (2)	
H4	0.3894	0.5489	0.8632	0.03*	
C5	0.6459 (3)	0.43752 (15)	0.92384 (5)	0.0242 (2)	
C6	0.6377 (4)	0.24623 (17)	0.93348 (5)	0.0293 (3)	
H6	0.5586	0.2064	0.9682	0.035*	
C7	0.5554 (5)	0.56563 (19)	0.96737 (5)	0.0372 (3)	
H7A	0.678	0.6721	0.9638	0.056*	
H7B	0.5964	0.5128	1.0015	0.056*	
H7C	0.3342	0.5936	0.9645	0.056*	
C8	0.7980 (6)	0.3240 (2)	0.73390 (6)	0.0452 (4)	
H8A	0.6217	0.2477	0.7264	0.068*	
H8B	0.9791	0.2537	0.7433	0.068*	
H8C	0.8461	0.3938	0.7029	0.068*	
O1	0.5405 (5)	−0.03468 (14)	0.89674 (5)	0.0594 (5)	
O2	0.8156 (3)	0.65036 (13)	0.85837 (4)	0.0392 (3)	
H2A	0.735	0.7419	0.8696	0.059*	
O3	0.7187 (4)	0.43952 (13)	0.77758 (4)	0.0379 (3)	
O4	0.9367 (3)	0.34098 (15)	0.93283 (4)	0.0350 (2)	

### Atomic displacement parameters (Å^2^)

**Table:**

	*U*^11^	*U*^22^	*U*^33^	*U*^12^	*U*^13^	*U*^23^
C1	0.0486 (8)	0.0182 (5)	0.0379 (6)	0.0011 (5)	0.0071 (7)	−0.0006 (4)
C2	0.0484 (8)	0.0210 (5)	0.0318 (6)	−0.0004 (5)	0.0055 (6)	−0.0066 (4)
C3	0.0325 (6)	0.0226 (5)	0.0253 (5)	0.0013 (5)	0.0029 (5)	−0.0029 (4)
C4	0.0297 (5)	0.0174 (4)	0.0272 (5)	0.0020 (4)	0.0042 (4)	−0.0011 (4)
C5	0.0254 (5)	0.0211 (5)	0.0261 (5)	−0.0009 (4)	0.0018 (4)	−0.0029 (4)
C6	0.0360 (6)	0.0231 (5)	0.0289 (5)	0.0008 (5)	0.0029 (5)	0.0024 (4)
C7	0.0521 (9)	0.0286 (6)	0.0310 (6)	−0.0041 (6)	0.0082 (6)	−0.0093 (5)
C8	0.0645 (11)	0.0422 (8)	0.0290 (6)	0.0011 (9)	0.0103 (7)	−0.0080 (6)
O1	0.1057 (14)	0.0182 (4)	0.0545 (7)	−0.0065 (6)	0.0200 (9)	0.0002 (4)
O2	0.0545 (7)	0.0199 (4)	0.0431 (5)	−0.0081 (4)	0.0158 (5)	−0.0024 (4)
O3	0.0593 (7)	0.0294 (4)	0.0250 (4)	0.0029 (5)	0.0074 (5)	−0.0019 (3)
O4	0.0280 (5)	0.0368 (5)	0.0400 (5)	0.0022 (4)	−0.0047 (4)	0.0019 (4)

### Geometric parameters (Å, °)

**Table:**

C1—O1	1.2292 (17)	C5—C7	1.5074 (17)
C1—C2	1.444 (2)	C6—O4	1.4506 (18)
C1—C6	1.4924 (19)	C6—H6	0.98
C2—C3	1.3503 (17)	C7—H7A	0.96
C2—H2	0.93	C7—H7B	0.96
C3—O3	1.3379 (15)	C7—H7C	0.96
C3—C4	1.5113 (16)	C8—O3	1.4393 (17)
C4—O2	1.4162 (17)	C8—H8A	0.96
C4—C5	1.5162 (17)	C8—H8B	0.96
C4—H4	0.98	C8—H8C	0.96
C5—O4	1.4448 (16)	O2—H2A	0.82
C5—C6	1.4640 (18)		
			
O1—C1—C2	123.24 (14)	O4—C6—C5	59.43 (9)
O1—C1—C6	118.88 (14)	O4—C6—C1	112.79 (12)
C2—C1—C6	117.85 (12)	C5—C6—C1	119.79 (11)
C3—C2—C1	121.21 (11)	O4—C6—H6	117.2
C3—C2—H2	119.4	C5—C6—H6	117.2
C1—C2—H2	119.4	C1—C6—H6	117.2
O3—C3—C2	124.38 (11)	C5—C7—H7A	109.5
O3—C3—C4	111.41 (10)	C5—C7—H7B	109.5
C2—C3—C4	124.08 (11)	H7A—C7—H7B	109.5
O2—C4—C3	108.50 (10)	C5—C7—H7C	109.5
O2—C4—C5	110.21 (11)	H7A—C7—H7C	109.5
C3—C4—C5	113.09 (10)	H7B—C7—H7C	109.5
O2—C4—H4	108.3	O3—C8—H8A	109.5
C3—C4—H4	108.3	O3—C8—H8B	109.5
C5—C4—H4	108.3	H8A—C8—H8B	109.5
O4—C5—C6	59.82 (9)	O3—C8—H8C	109.5
O4—C5—C7	115.19 (12)	H8A—C8—H8C	109.5
C6—C5—C7	120.44 (11)	H8B—C8—H8C	109.5
O4—C5—C4	114.18 (10)	C4—O2—H2A	109.5
C6—C5—C4	119.29 (10)	C3—O3—C8	118.11 (11)
C7—C5—C4	115.41 (11)	C5—O4—C6	60.75 (8)
			
O1—C1—C2—C3	168.6 (2)	C7—C5—C6—O4	−103.26 (15)
C6—C1—C2—C3	−13.3 (3)	C4—C5—C6—O4	102.54 (13)
C1—C2—C3—O3	179.30 (16)	O4—C5—C6—C1	−100.39 (15)
C1—C2—C3—C4	−5.2 (3)	C7—C5—C6—C1	156.36 (15)
O3—C3—C4—O2	−40.34 (16)	C4—C5—C6—C1	2.2 (2)
C2—C3—C4—O2	143.68 (16)	O1—C1—C6—O4	125.98 (19)
O3—C3—C4—C5	−162.94 (13)	C2—C1—C6—O4	−52.22 (19)
C2—C3—C4—C5	21.1 (2)	O1—C1—C6—C5	−167.29 (19)
O2—C4—C5—O4	−72.54 (13)	C2—C1—C6—C5	14.5 (2)
C3—C4—C5—O4	49.10 (15)	C2—C3—O3—C8	−7.4 (3)
O2—C4—C5—C6	−140.21 (13)	C4—C3—O3—C8	176.65 (16)
C3—C4—C5—C6	−18.57 (18)	C7—C5—O4—C6	111.98 (13)
O2—C4—C5—C7	64.33 (16)	C4—C5—O4—C6	−111.05 (12)
C3—C4—C5—C7	−174.03 (13)	C1—C6—O4—C5	112.19 (13)

### Hydrogen-bond geometry (Å, °)

**Table:**

*D*—H···*A*	*D*—H	H···*A*	*D*···*A*	*D*—H···*A*
O2—H2A···O1^i^	0.82	1.99	2.8148 (17)	180.
C4—H4···O2^ii^	0.98	2.54	3.521 (2)	176.
C6—H6···O4^iii^	0.98	2.56	3.5208 (17)	167.

Symmetry codes: (i) *x*, *y*+1, *z*; (ii) *x*−1, *y*, *z*; (iii) *x*−1/2, −*y*+1/2, −*z*+2.
